# Sedentary Behaviour in Hospitalised Older People: A Scoping Review

**DOI:** 10.3390/ijerph17249359

**Published:** 2020-12-14

**Authors:** Unyime Jasper, Lalit Yadav, Joanne Dollard, Agathe Daria Jadczak, Solomon Yu, Renuka Visvanathan

**Affiliations:** 1 Adelaide Geriatrics Training and Research with Aged Care (G-TRAC) Centre, Adelaide Medical School, Faculty of Health and Medical Sciences, University of Adelaide, Adelaide, SA 5011, Australia; joanne.dollard@adelaide.edu.au (J.D.); agathedaria.jadczak@adelaide.edu.au (A.D.J.); solomon.yu@adelaide.edu.au (S.Y.); renuka.visvanathan@adelaide.edu.au (R.V.); 2 National Health and Medical Research Council Centre of Research Excellence, Frailty and Healthy Ageing, University of Adelaide, Adelaide, SA 5011, Australia; lalit.yadav@adelaide.edu.au; 3 Aged and Extended Care Services and Basil Hetzel Institute, The Queen Elizabeth Hospital, Central Adelaide Local Health Network, Adelaide, SA 5011, Australia

**Keywords:** scoping review, sedentary behaviour, older people, hospital, in-patient rehabilitation

## Abstract

*Background:* Sedentary behaviour (SB) can delay hospitalised older adults’ recovery from acute illness and injuries. Currently, there is no synthesis of evidence on SB among hospitalised older people. This scoping review aimed to identify and map existing literature on key aspects of SB among hospitalised older adults, including the prevalence, measurement and intervention strategies for SB and sedentary behaviour bouts (SBBs) as well as healthcare professionals, patients and carers’ perspectives on interventions. *Methods and analysis:* Several electronic databases were searched between January 2001 and September 2020. The Joanna Briggs Institute (JBI) framework was used to conduct this scoping review. *Results:* Out of 1824 articles, 21 were included comprising 16 observational studies, 3 randomised controlled trials, 1 comparative study, and 1 phase-1 dose-response study. The sample size ranged from 13 to 393, with all 1435 participants community-dwelling before hospitalisation. Only two studies focused on measuring SB and SBBs as a primary outcome, with others (n = 19) reporting SB and SBB as a sub-set of physical activity (PA). Older adults spent an average of 86.5%/day (20.8 h) sedentary. Most studies (n = 15 out of 21) measured SB and SBB using objective tools. *Conclusion:* Hospitalised older people spent most of their waking hours sedentary. Studies explicitly focused on SB and SBB are lacking, and the perspectives of patients, carers and healthcare professionals are not clarified. Future hospital-based studies should focus on interventions to reduce SB and SBB, and the perspectives of healthcare professionals, patients and carers’ taken into account.

## 1. Introduction

Hospitalised older people are at risk of developing iatrogenic conditions independent of the primary reason for admission [1]. Some of these include functional decline and increased frailty [2], resulting in hospital-acquired disability (HAD) [3]. One main reason for HAD is sedentary behaviour (SB) [4], which has been shown to result in increased loss of muscle mass and function [5,6]. The effects of SB during hospital admission persist post-discharge [7], with previously active older people more sedentary at home post-discharge as a result of HAD [8]. In hospitals, when older people conduct activities such as sitting, lying and reclining whilst awake, they are said to be exhibiting SB [9]. Sedentary behaviour bout (SBB) [9] on the other hand relates to the extent people are sedentary and is said to be occurring when less than 100 steps/min are made for a period of one minute or more. Being aware of the extent of SB and SBB is an essential first step when highlighting the need for effective interventions aimed at reducing SB and SBB in hospitalised older people.

Health professionals are embracing campaigns such as #EndPJparalysis with the hope of tackling the epidemic of sedentary behaviour (SB) in hospitals, encouraging patients to exchange hospital gowns or pyjamas (“PJ”) for street clothes and to get moving [10]. Despite the roll-out of such campaigns, no reviews have explored current evidence related to SB in hospitalised older adults. Describing the state of current evidence on SB could enable researchers and practitioners to develop strategies to prevent, decrease and alleviate the undesired consequences of SB for hospitalised older adults and their caregivers.

This scoping review aimed to identify and map (classify, organise and synthesise) relevant peer-reviewed articles on SB and SBB in hospitalised older adults. A scoping review methodology was used due to the broad nature of the topic and to enable the identification of currently available evidence to guide the development of future research studies.

## 2. Methodology

According to our previously published protocol paper [11], we followed the Joanna Briggs Institute (JBI) framework for scoping reviews [12]. The findings of this review are reported according to the Preferred Reporting Items for Systematic Reviews and Meta-Analysis extension for Scoping Reviews (PRISMA-ScR) checklist [13] (Supplementary Materials S1).

### 2.1. Population

This review included studies involving participants aged ≥65 years (or mean age of ≥65 years).

### 2.2. Concept

Studies considering various characteristics of SB and SBB including prevalence, assessment tools (objective or subjective), interventions as well as perspectives of healthcare professionals, patients and carers regarding intervention, were included.

### 2.3. Context

Settings considered were in-patient hospital and rehabilitation. Community settings, nursing homes or residential aged care were excluded. Studies from a broad range of research methodologies covering cross-sectional, observational, interventional, qualitative and mixed methods studies were included.

Articles from January 2001 to September 2020 were searched using Medical Subject Heading (MeSH) terms and keywords, such as sedentary behaviour, hospital and aged (Supplementary Materials S2). The following electronic databases were searched: PEDro, PubMed, Web of Science, MEDLINE Ovid, Cochrane, Scopus, Cumulative Index to Nursing and Allied Health Literature (CINAHL), PsychInfo, Embase, Ageline, JBI and clinical trials registries for interventions.

### 2.4. Study Selection

Articles were screened and included using a two-step approach. Two independent reviewers (U.J. and L.Y.) screened the abstracts. Then, the full text of relevant articles was reviewed for inclusion (Supplementary Materials S3). Disagreements were clarified by discussing the differences, and a third reviewer (J.D.) was invited if a consensus could not be reached.

### 2.5. Data Extraction

Two independent reviewers (U.J. and L.Y.) extracted data using a data extraction tool developed according to the aims of the study (Supplementary Materials S4). Data extracted included: number of participants, duration of SB and SBBs, assessment of SB and SBBs, study design, study population and intervention details.

### 2.6. Presentation of the Results/Data Mapping

Findings are presented as a narrative summary and in a tabular format.

## 3. Results

### 3.1. Characteristics of Included Studies

After removing duplicates, 58 articles were considered for full-text review but only 21 peer-reviewed publications were finally deemed eligible (Table 1), with five additional articles were identified from secondary reference searches [14,15,16,17,18]. The majority of studies (19 out of 21) were conducted within the last eleven years (i.e., 2010–2020). Seven studies each were conducted in Australia [14,15,16,17,18,19,20] and Europe [21,22,23,24,25,26,27] respectively, three in the United States [28,29,30], two in Brazil [31,32] and one each in Israel [33], and Canada [34].

### 3.2. Study Settings

Fourteen studies recruited participants from acute wards [14,15,19,20,21,22,23,25,26,27,28,29,30,31,32,33], five from in-patient rehabilitation units [16,17,18,24,34] and two from geriatric psychiatry settings [25,26]. Of the 14 studies conducted in acute wards, four focused on patients with stroke [14,21,22,23], four on patients with various medical conditions [19,20,28,29] and one each on patients with respiratory disease [31], heart failure [30], chronic obstructive pulmonary disease [32], idiopathic pulmonary fibrosis [33], hip fracture [15] and abdominal surgery [27] respectively. The majority of studies were cross-sectional [14,15,16,20,21,22,23,24,25,26,28,29,30,33,34] with only one longitudinal study [32] and four smaller trials [17,18,27,31].

### 3.3. Participants

The mean age of participants ranged from 66 to 79 years, with all participants community-dwelling before hospitalisation. A total of 1435 participants were included in the 21 studies deemed eligible. The sample size ranged from 13 to 393 participants. Four studies included more than 100 participants, and all were cross-sectional and utilised behavioural mapping with three in acute wards [20,21,22,24] and one in in-patient rehabilitation [24]. Two studies [25,26] included participants with cognitive impairment, nine studies excluded participants with cognitive impairment [14,15,16,17,18,19,24,29,30] while others did not mention whether participants with cognitive impairment were included [20,21,22,23,27,28,31,32,33,34].

### 3.4. SB and SBB Measurements

Only two studies measured SB and SBB as a primary outcome [24,34] (Table 1). Both studies focused on patients with stroke with varying sample sizes (i.e., n = 19 and n = 104) and used different methods to investigate SB and SBB. Barrett et al. [34] met the current definition of SB by excluding sleep time, measuring SB and SBB over seven days but with a smaller sample size of 19. The larger study [n = 104] by Sjoholm et al. [24], explored SB and SBB using behavioural mapping for 9 h (8 am to 5 pm) on one day.

The other nineteen studies [14,15,16,17,18,19,20,21,22,23,25,26,27,28,29,30,31,32,33] measured PA as a primary outcome, and data reproducing SB and SBBs was extrapolated from results based on time spent lying or sitting in a bed and chair. Therefore none of the studies met the definition of SB and SBB. Measurement duration ranged from 9 h (8 am to 5 pm) to 30 days.

Ten tools were used to measure SB and SBBs (Table 2). A total of thirteen studies used objective tools only [15,16,17,18,23,25,26,27,29,30,31,32,34], two studies used a combination of objective and subjective methods [14,28], while six studies used subjective tools only [19,20,21,22,24,33].

Subjective methods in the majority (5 out of 8 studies) utilised the behavioural mapping technique alone [19,20,21,22,24]. Of the remaining, one study used the behavioural mapping technique with an objective tool [14], one study used the International Physical Activity Questionnaire (IPAQ) [33] and one study used direct observation [28]. Behavioural mapping has been validated to measure PA, as well as sitting and lying in hospitalised patients with stroke, but has not been validated as a method to measure SB or SBB [14].

The only wearable devices validated for the measurement of SB and SBB in hospital are ActivPAL and PAL2 [35,36]. ActivPAL was the most commonly (6 out of 8 studies) used of the two wearable devices. Six studies utilised ActivPAL and PAL2 as the sole measurement strategy [15,16,17,18,23,27], or in combination with another wearable device called Tractivity [30], or in combination with behavioural mapping [14].

Three studies reported on the acceptability of wearable devices (Table 2), two relating to the PAL 2 sensor (attached to thigh) [14,18] and one to the Usense sensor (attached to lower back) [26]. The two studies with PAL2 worn on the lower thigh reported acceptability of 81% [18] and 90%, respectively [14]. Primary reasons for declining or removing a sensor in all three studies were discomfort [14,18,26] with other reasons such as not wanting to be monitored, lack of interest, inconvenience, anxiety and interference with toileting/indwelling catheter reported in one study [18].

### 3.5. Proportion of SB and SBB

Overall, the mean duration of SB from all the studies was 86.5%/day (20.8 h)

### 3.6. Studies with SB and/or SBB as Primary Outcome

Sjoholm et al. revealed that participants were sedentary for 74% (6.2 h) over nine hours between 8 am and 5 pm [24] (Table 1). In this study, SBB of >1 h was noted among 54% of participants; the average SBB was 38 min (10–490 min), and the mean number of SBBs per patient was 9.9.

The smaller study by Barrett et al. [34] that measured SB over seven days reported participants were sedentary for 85.6% of the observed duration (12. 8 h/day). There was no significant difference in participants SB between admission and discharge, but an increase in participants SB on the weekend to 89.8% (13.5 h/day). Including sleep periods, this study reported average SBBs of 5.31 h (318.8 ± 219.9 min) [34].

### 3.7. Studies Where SB and/or SBB Were Extrapolated

As shown in Table 1, the 11 studies that used wearable devices reported a relatively higher mean prevalence of SB (85.6–91.6%) [15,16,17,18,23,25,26,29,30,32,34] compared to studies that utilised subjective behavioural mapping techniques (74.0–87.3%) [14,20,21,22,24]. Studies that utilised wearables did not report how they handled data on sleep time [15,16,17,18,23,25,26,27,28,29,30,31,32] and studies that utilised behavioural mapping did not report how they handled sleep time data [14,19,20,21,22,24]. Only one study using behavioural mapping reported SB prevalence similar to that noted for wearable devices (94.0%) [16], most likely due to capturing all forms of sitting as SB, compared to other behavioural mapping based studies that interpreted sitting unsupported out of bed and rolling to sit up on the bed as PA.

A study among 58 patients with stroke using ActivPAL [23] demonstrated that SBB increased by 4.32 min daily over seven days among those with severe stroke and older participants. The study included sleep time and excluded patients with dementia

### 3.8. Perspectives of Stakeholders about Interventions

Despite our comprehensive search strategy and broad inclusion criteria, we did not identify studies reporting stakeholders (healthcare practitioners), end-users (patients and their formal or informal carers) perspectives on interventions to reduce SB or SBB within hospital settings.

### 3.9. Intervention Strategies for SB and SBB

No intervention studies specifically focused on reducing SB and SBB as the primary aim.

## 4. Discussion

Findings from this study revealed that while hospitalised older people spent most of their waking hours sedentary, the prevalence of SB and SBB cannot be established due to poor adherence to current definitions of SB and SBB and a lack of consensus on how best to measure SB and SBB within hospital and in-patient rehabilitation settings. Additionally, no intervention studies have focused explicitly on reducing SB or SBB as primary aims, and no study has sought the perspectives, attitudes and experiences of patients, carers and health professionals, especially crucial if translatable interventions are to be developed. Thus, the findings from this review suggest there remains a need for developing hospital-based interventions to reduce SB and SBBs by taking into account the perspectives of healthcare professionals, older patients and their carers.

Only two studies focused primarily on SB and SBBs, revealing a gap in knowledge on the prevalence of and interventions for SB and SBBs among hospitalised older adults. Possibly, the non-recognition of SB and SBBs as distinct risk-factors from PA in the development of geriatric-specific poor health outcomes [37] was a reason for the small number of hospital studies focused on SB. We, therefore, have limited understanding of the prevalence, timing, frequency and determinants of SB in hospitalised older adults. In recent years there has been an increasing trend in exploring the extent of SB in older people, especially in the community. For example, Stamatakis et al. [38] found that 67% of older people spend <8.5 h sedentary while awake using an objective measure, while a combined analysis of seven studies that explored self-reported sitting time found that almost 60% of older people were sedentary for >4 h/day [39]. Thus, there is a need to explore the extent of SB and SBBs in hospital, to generate evidence and guide the development of appropriate interventions.

Currently, no hospital-based intervention studies have focused on reducing SB and SBBs in older people. The four intervention studies in this review focused on PA [17,18,27,31] and reported SB as secondary outcomes, a possible reason interventions had no significant impact on SB. Interventions exclusively targeting SB and SBBs are required to decrease SB. Purely advising patients to increase PA [40] may not address SB (and SBB) because a person might be active for a few minutes but spend the rest of the day sedentary. A better approach could be to specifically intervene to reduce SB whilst also increasing activity [41] especially for hospitalised older people who may lack the fitness to achieve moderate to vigorous levels of PA. Activities that interrupt periods of SB could include sit-to-stands, walking to the lounge room to socialise with other patients or relatives and walking to the restroom rather than using a commode.

With few studies exploring acceptability, it is unclear if older people are comfortable wearing devices or being observed. Interventions must be acceptable to end-users if there is to be adherence to and translation of the intervention into healthcare [42]. Thus, understanding the perspectives of stakeholders and end-users regarding interventions on ubiquitous health issues as SB is critical to reducing SB among hospitalised older people successfully. With better understanding and integration of the perspectives of end-users and stakeholders, investigators can design SB interventions that are effective and acceptable to both groups [42]. The lack of end-users perspectives is likely contributing to the dearth of intervention studies explicitly focused on reducing SB and SBB in hospitalised older people.

There is a lack of validated wearables and subjective tools for measuring SB in hospitalised older people. A previous systematic review has reported a dearth of valid and reliable objective tools to measure SB in older adults [43]. Regarding subjective measures, the behavioural mapping technique is validated to describe activity in older patients with stroke [44] while the IPAQ-SF is unsuitable for assessing SB in older adults [45]. For objective tools, only PAL2 has been validated to measure SB in natural hospital activities [35], while ActivPAL has only been validated to measure SB during a controlled test protocol of 1 h [36]. There are concerns that for longer durations in conventional hospital settings, the measurement error may be larger, and the validity of the results may be different from that in the controlled protocol [36]. It is suggested that there is a need for validity and reliability studies focused on older people where the required wear time, as well as position of devices for objective methods, are better clarified [43].

Consensus on the best methods to measure SB and SBB in hospitals is important. A previous systematic review exploring older people’s SB found discrepancies between subjective and objective measures [39]. The behavioural mapping technique, for example, classifies SB (rolling in bed, transfers and sitting in bed/chair) as PA and its method of calculating SBBs (as time spent in SB/number of transitions from lying to sitting) underestimates it. Data capture, be it with subjective methods or wearable devices, should focus on awake periods and exclude sleep time. The heterogeneity of methods with many studies failing to meet or to report if they had met currently accepted definitions makes comparisons between studies difficult. Therefore, the true extent of SB in hospitals whilst high [86.5%/day (20.8 h)] is currently not known.

## 5. Strengths and Limitations

This scoping review has some limitations. The inclusion of studies with a mean age ≥65 years may have resulted in the exclusion of findings from studies involving younger cohorts. A focus on English only studies might have resulted in missing important research in other languages.

## 6. Conclusions

This scoping review has highlighted the need for hospital-based studies focused on SB and SBB. Importantly, there is a need to better understand the perspectives of healthcare professionals, carers and patients, as this would support the design and investigation of translatable interventions to reduce SB and SBB in hospitals. While older patients spend most of their waking hours sedentary, the prevalence of SB and SBB in hospitalised older people is unclear due to poor adherence to the current definitions and a lack of consensus on how best to measure SB and SBB in hospital. Future hospital-based studies should focus on designing context-specific interventions to reduce SB and SBBs and prioritise seeking healthcare professionals, patients and carers’ perspectives of SB and SBB interventions to aid subsequent intervention design and implementation. This may improve adherence to and translatability of such interventions into clinical practice. The opportunity for further research on this topic is extensive and crucial if this health issue is to be addressed.

## Figures and Tables

**Table 1:** Summary of included studies.

Author/Year Country	Condition/Setting/Ward Type	Study Design and Recruitment	n (n Women)	Mean Age (SD)	Primary Outcome	Definition of SB Used in Study	Measurement	Duration of Monitoring	Daily Duration of SB (%)	Mean Sedentary Behaviour Bouts (SBB)
**Studies measuring SB and SBB**										
Sjöholm (2014) [24] Sweden	Stroke/Rehabilitation/Stroke rehabilitation clinic	Cross-sectional Cognitively intact only; ≥18 years	104 (49)	70.3 (14.4)	Sedentary time	SB: Time spent lying and sitting supported in bed (sitting unsupported in bed measured as physically active); included sleep time as SB SBB: time spent in SB/number of transitions from lying to sitting	Subjective (behavioural mapping)	9 h (8 am–5 pm)	6 h 20 min (74.0%) of observed time	1.23 (1.2) h
Barrett (2018) [34] Canada	Stroke/Rehabilitation/Stroke rehabilitation unit	Cross-sectional Convenience sampling; first stroke; ≥18 years	19 (7)	68.2 (9.8)	Sedentary time	SB: Time spent lying and sitting; sleep time excluded SBB: Long blocks (≥60 min) of time in which participants classified as sedentary	Objective (Actiheart)	7 days following admission and 7 days before discharge	12 h 45 min (85.6%) during weekday 13 h 30 min during the weekend (89.8%)	2.23 (1.3) h
**Studies extrapolating SB from PA**										
Askim (2011) [21] Norway	Stroke/Acute hospital/Stroke unit	Cross-sectional Time of stroke onset < 14 days	117 (52)	78.7 (9.2)	Motor Activity	Time spent lying and sitting supported in bed as part of PA results (sitting unsupported in bed measured as physically active); no report on sleep time SBB: Not reported	Subjective (behavioural mapping)	9 h (8 am–5 pm)	6 h 54 min (76.7%) of observed time	Not reported
Hokstad (2015) [22] Norway	Stroke/Acute hospital/Stroke unit	Cross-sectional Time of stroke onset < 14 days; aged ≥ 18 years	393 (204)	76.7 (11.2)	Motor activity	Time spent lying and sitting supported in bed as part of PA results (sitting unsupported in bed measured as physically active); no report on sleep time SBB: Not reported	Subjective (behavioural mapping)	Two different days or two consecutive days	20 h 50 min (87.3%)	Not reported
Cattanach (2014) [19] Australia	Various conditions/Acute hospital/Medical ward	Cross-sectional Convenience sampling; ≥18 years; Cognition intact	24 (14)	77 (2.0)	Physical activity	Time spent lying as part of PA results (sitting out of bed measured as physically active); no report on sleep time SBB: Not reported	Subjective (behavioural mapping)	9 h (8 am–5 pm)	8 h 28 min (94.0%) of observed time	Not reported
Kuys (2012) [20] Australia	Various conditions/Acute hospital/Internal medicine unit	Cross-sectional Convenience sampling; admitted for at least 2 days; ≥18 years	102 (38)	66.3 (19.6)	Activity level	Time spent lying and sitting as part of PA results; no report on sleep time SBB: Not reported	Subjective (behavioural mapping)	9 h (8 am–5 pm)	5 h 25 min (58.3%) of observed time	Not reported
Vainshelboim (2018) [33] Isreal	Idiopathic Pulmonary Fibrosis (IPF)/Acute hospital/pulmonary ward	Cross-sectional Convenience sampling; met criteria for IPF diagnosis; stable 3–6 months previously	34 (12)	Median 68 (IQR = 50–81)	Sitting/walking	Time spent lying and sitting as part of PA results; no report on sleep time SBB: Not reported	Subjective (IPAQ-sf)	Past week	Median (IQR) = 8.5 (2–15) h	Not reported
Kramer (2013) [14] Australia	Stroke/Acute hospital/Acute stroke ward	Cross-sectional Time of stroke onset <14 days; included cognitive impaired; ≥18 years	20 (10)	Median 80 (IQR = 76.5–83.5)	Activity after stroke	Time spent lying and sitting as part of PA results; no report on sleep time SBB: Not reported	Objective (PAL 2) Subjective (behavioural mapping)	9 h (8 am–5 pm)	20 h 53 min (87.0%) PAL2 18 h 58 min (79.0%) for behavioural mapping)	Not reported
Brown (2008) [28] United States	Various conditions/Acute hospital/Medical wards	Cross-sectional Convenience sampling; medical problems; no isolation precaution; ≥65 years	45 (0)	73.9 (6.6)	Mobility level	Time spent lying and sitting as part of PA results; no report on sleep time SBB: Not reported	Objective (wireless monitor) Subjective (direct observation)	7 days or until discharge for Wireless monitor; two consecutive days for direct observation	17 h 40 min (73.7%) 17 h 3 min (71.3%)	Not reported
Norvang (2018) [23] Norway	Stroke/Acute hospital/Stroke	Cross-sectional Time of stroke onset < 7 days; first-ever/recurrent stroke	58 (31)	75.1 (12)	Time upright, sitting and lying	Time spent lying and sitting as part of PA results; no report on sleep time SBB: time spent in sitting/number of transitions from lying to sitting	Objective (ActivPAL)	7 days	22 h 10 min (91.6%)	Increased 4.32 min daily
Brown (2009) [29] United States	Various conditions/Acute hospital/Medical wards	Cross-sectional Convenience sampling; medical problems; no isolation precaution; ≥65 years	45 (0)	74 (6.5)	Mobility level	Time spent lying and sitting as part of PA results; no report on sleep time SBB: Not reported	Objective (wireless monitor)	7 days	20 h (83.3%)	Not reported
Raymond (2018) [18] Australia	Various conditions/Rehabilitation/Not stated	Randomised controlled trial Randomisation sequence; intact cognition; ≥65 years	30 (not stated)	83.6 (6.9)	Physical activity	Time spent lying and sitting as part of PA results; no report on sleep time SBB: Not reported	Objective (PAL 2)	5 days, overlapping a weekend	Median (IQR) 16.6 (12.9–19.0) h/day for control group 14.4 (12.8–14.9) h/day for intervention group	Not reported
Fleiner (2019) [25] Germany	Dementia/Acute hospital/Geriatric psychiatry	Cross-sectional Convenience sampling; TUG without assistance; ≥20 in the MMSE#; Dementia with ICD-10-criteria	64 (30)	81 (6.2)	Physical activity	Time spent lying, sitting and standing sedentary as part of PA results; no report on sleep time SBB: Not reported	Objective (hybrid motion sensor)	3 days	21 h 50 min (90%)	Not reported
Fleiner (2016) [26] Germany	Dementia/Acute hospital/Geriatric psychiatry	Cross-sectional Convenience sampling; TUG without assistance; ≥20 in the MMSE#; Dementia with ICD-10-criteria	45 (26)	79 (7)	Mobility inactivity	Time spent lying, sitting and standing sedentary as part of PA results; no report on sleep time SBB: Not reported	Objective (hybrid motion sensor)	3 days	20 h 35 min (85.5%)	Not reported
Davenport (2014) [15] Australia	Hip fracture/Acute hospital/Orthopaedic ward	Cross-sectional Convenience sampling; from home or low-level facility, walking independently/assistance; no cognitive impairment; hip fracture	20 (18)	79.1 (9.3)	Physical activity	Time spent lying and sitting as part of PA results; no report on sleep time SBB: Not reported	Objective (activPAL)	7 days	23 h 44 min (98.9%)	Not reported
Taylor (2015) [17] Australia	Hip fracture/Rehabilitation/Not stated	Phase 1 dose-response trial Convenience sampling; walking independently/assistance; no cognitive impairment; hip fracture	13 (9)	81.3 (10.2)	Physical activity	Time spent lying and sitting as part of PA results; no report on sleep time SBB: Not reported	Objective (activPAL)	5 days	22 h 35 min (94.2%)	Not reported
Peiris (2013) [16] Australia	Lower limb fractures (not hip fracture)/Rehabilitation/Not stated	Cross-sectional ≥18 years; admitted for rehabilitation; able to walk independently; cognitively alert	54 (40)	74 (11)	Physical activity	Time spent lying and sitting as part of PA results; no report on sleep time SBB: Not reported	Objective (activPAL)	5 days	23 h (95.8%)	Not reported
Porserud (2019) [27] Sweden	Abdominal surgery due to colorectal, urinary bladder or ovarian cancer/Acute hospital/Surgical ward	Non-randomised controlled trial (cluster) Randomly allocated according to date for surgery; parallel groups; ≥18 years	67 (32) intervention group; 66 (34) control group	68.1 (12.3)	Mobility level after abdominal surgery due to cancer	Time spent lying and sitting as part of PA results; no report on sleep time SBB: Not reported	Objective (activPAL)	5 days	21 h (90%) intervention group; 21 h 30 min (91%) control group	Not reported
Moreno (2019) [31] Brazil	Respiratory illness/Acute care/Respiratory ward	Randomised clinical trial 1:1 allocation using a randomisation website; ≥60 years; hospitalized for any clinical condition for 48 h; able to mobilise without professional or carer assistance	33 (17) control 35 (11) intervention	69 (7)	Physical activity during hospitalisation	Time spent lying and sitting as part of PA results; no report on sleep time SBB: Not reported	Objective (ActiGraph)	24h/day until hospital discharge	15 h 12 min (63%) intervention group; 16 h 30 min (68%) control group	Not reported
Floegel (2018) 30] United States	Heart failure/Acute hospital/General medical unit	Cross-sectional Convenience sampling; ≥62 years of age; admitted for heart failure; able to walk independently; excluded cognitively impaired	27 (14)	78 (9.8)	Posture/ambulation	Time spent lying and sitting as part of PA results; no report on sleep time SBB: Not reported	Objective (activPAL/Tractivity)	30 days	22 h 40 min (93.3%)	Not reported
Borges (2012) [32] Brazil	Chronic Obstructive Pulmonary Disease (COPD)/Acute hospital/not stated	Longitudinal Convenience sampling; met criteria for COPD diagnosis	20 (6)	68.6 (10.7)	Physical activity	Time spent lying and sitting as part of PA results; no report on sleep time SBB: Not reported	Objective (DynaPort movemonitor)	12h/day (from 08:00 to 20:00) on 3rd & 4th days of hospitalisation	20 h 48 min (86.7%)	Not reported

SB = sedentary behaviour; PA = physical activity; SBB = sedentary behaviour bout.

**Table 2:** Characteristics of tools measuring sedentary behaviour (SB) and sedentary behaviour bouts (SBBs) among older patients in hospital.

Author/Year Country	Method	Device/Tool	Site Worn (Secured with)	Validity to Measure SB in Hospital	Acceptability of Tool
Norvang (2018) [23] Norway	Objective	Triaxial ActivPAL	Sternum and mid-thigh	Yes	Not reported
Barrett (2018) [34] Canada	Objective	Actiheart	Over the heart	No	Not reported
Brown (2009) [29] United states	Objective	Wireless monitor	Ipsilateral thigh and ankle (using a large bandage)	No [No longer in production]	Not reported
Raymond (2018) [18] Australia	Objective	PAL2	Lower thigh	Yes	19% (n = 30) agreed to wear the PAL2 device 66.7% (n = 104) of participants declined due to lack of interest or not wanting to be monitored; 23% (n = 7) removed the PAL2 before the final day [discomfort (n = 2), anxiety (n = 2) and interference with toileting/indwelling catheter (n = 3)]
Fleiner (2019) [25] Germany	Objective	Hybrid motion sensor (uSense)	Lower back	No	Not reported
Fleiner (2016) [26] Germany	Objective	Hybrid motion sensor (uSense)	Lower back	No	2.2% (n = 1) rejected sensor; 8.9% (n = 4) removed sensor prior 72h assessment
Davenport (2014) [15] Australia	Objective	Uniaxial ActivPAL	Not stated	Yes	Not reported
Taylor (2015) [17] Australia	Objective	Uniaxial ActivPAL	Not stated	Yes	Not reported
Peiris (2013) [16] Australia	Objective	Uniaxial ActivPAL	mMid-thigh of unaffected leg	Yes	Not reported
Floegel (2018) [30] United States	Objective	Uniaxial ActivPAL and Tractivity accelerometer	ActivPAL on rib cage (1 inch below nipple line) and mid-thigh. Tractivity right ankle	Yes for ActivPAL	Not reported
Borges (2012) [32] Brazil	Objective	DynaPort Movemonitor	Lower back (2nd lumbar vertebrae)	No	Not reported
Porserud (2019) [27] Sweden	Objective	Triaxial ActivPAL	Below the collar bone and mid-thigh	Yes	Not reported
Moreno (2019) [31] Brazil	Objective	ActiGraph	Wrist of dominant hand	No	Not reported
Brown (2008) (28) United states	Objective/subjective	Wireless monitor/direct observation	Ipsilateral thigh and ankle (using a large bandage)	No [No longer in production]	Not reported
Kramer (2013) [14] Australia	Subjective Objective	PAL2 Behavioural mapping technique *	Lateral side of one leg (attached with two straps above and below the knee)	Yes No	No participant (0/20) took sensor off 3 reported that the straps used to attach the device to the leg were too tight and uncomfortable. Of participants, 8 (out of 20) responded to acceptability survey five out of eight strongly agreed, one participant was undecided and two disagreed with the statement “wearing the device on my leg was comfortable.”
Askim (2011) [21] Norway	Subjective	Behavioural mapping technique *	N/A (not applicable)	No	Not reported
Hokstad (2015) [22] Norway	Subjective	Behavioural mapping technique *		No	Not reported
Sjöholm (2014) [24] Sweden	Subjective	Behavioural mapping technique *		No	Not reported
Cattanach (2014) [19] Australia	Subjective	Behavioural mapping technique *		No	Not reported
Kuys (2012) [20] Australia	Subjective	Behavioural mapping technique *		No	Not reported
Vainshelboim (2018) [33] Isreal	Subjective	IPAQ-sf		No	Not reported

* The behavioural mapping technique is a method for observing activity in acute patients with stroke using 15 activities (e.g., sitting supported out of bed, roll and sit up, sitting supported in bed, eating with unaffected hand) (32) IPAQ: short-form International Physical Activity Questionnaire. SB = sedentary behaviour; PA = physical activity; SBB = sedentary behaviour bout.

## Supplementary Materials

The following are available online at https://www.mdpi.com/1660-4601/17/24/9359/s1, Supplementary S1: Preferred Reporting Items for Systematic reviews and Meta-Analyses extension for Scoping Reviews (PRISMA-ScR) Checklist; Supplementary S2: PRISMA-ScR flowchart; Supplementary S3: Reviewer: Date of data extraction; Supplementary S4: Search on PubMed
