# Supplementary material for: Sedentary Behaviour in Hospitalised Older People: A Scoping Review
Source: Int J Environ Res Public Health. 2020 Dec 14;17(24):9359. doi: 10.3390/ijerph17249359 (PMC7765084; doi:10.3390/ijerph17249359)
Supplement: Supplementary file 1 [file ijerph-17-09359-s001.zip › Supplementary Materials S2.docx]

Additional file 2

**PRISMA-ScR flowchart**

Eligible articles
(n = 16)

Full-text articles assessed for eligibility
(n = 58)

Title/abstracts screened
(n = 1824)

Duplicates removed
(n = 1726)

Articles identified through database searching
(n = 3550)

## Identification

Articles excluded
(n = 1766)

Conferences: 72

No data on SB: 1266

Language: 23

Age <65 years: 64

Community: 341

Outpatients: 205

No data on SB/SBBs: 1266

## Screening

## Eligibility

Full-text articles excluded (n = 42)

Age <65 years: 11

Outpatients: 14

No data on SB/SBBs: 17

Additional records identified through other sources
(n = 5)

## Included

Articles included in narrative summary
(n = 21)
