# Supplementary material for: Sedentary Behaviour in Hospitalised Older People: A Scoping Review
Source: Int J Environ Res Public Health. 2020 Dec 14;17(24):9359. doi: 10.3390/ijerph17249359 (PMC7765084; doi:10.3390/ijerph17249359)
Supplement: Supplementary file 1 [file ijerph-17-09359-s001.zip › Supplementary Materials S4.docx]

**Search on PubMed**

(((Aged[mh] OR Frail Elderly [mh])) AND (Physical activit*[tiab] OR Sedentary Behavior[mh])) AND (Hospitals[mh] OR Subacute care [mh] OR Rehabilitation[mh])
