# Supplementary material for: Sedentary Behaviour in Hospitalised Older People: A Scoping Review
Source: Int J Environ Res Public Health. 2020 Dec 14;17(24):9359. doi: 10.3390/ijerph17249359 (PMC7765084; doi:10.3390/ijerph17249359)
Supplement: Supplementary file 1 [file ijerph-17-09359-s001.zip › Supplementary Materials S3.docx]

Additional file 3 Data extraction form

Reviewer:

Date of data extraction:

**Data to be extracted Item Notes to reviewer**

Publication details

- Authors

Year

Article title

Journal, volume, issue, page numbers

Study design and details

- Setting (country; characteristics of hospital/acute care/rehabilitation)
- Sampling technique (convenience sampling; probability sampling)
- Sample size
- Details of study participants (age, sex, disease condition, etc.)
- Design (Survey; Randomised Control Trials (RCTs), etc.)
- Method of data collection (interviews; observation, etc.)
- Method of data analysis

Specific details of interest for the scoping review

- SB tools used (subjective; objective, etc.): name of tool,

abbreviation of the tool, details of the tool;

make of the tool (if objective)

- SBB tools used (subjective; objective, etc.): name of tool,

abbreviation of the tool, details of the tool;

make of the tool (if objective)

- Reported prevalence rates of SB and SBB
- Reported treatment modalities for SB and SBB
- Reported outcomes of the treatment of SB and SBB
- Geriatric specific outcomes reported as benefitting

from treatment of SB and SBB

- Reported patients, carers and health professionals perception

of intervention to reduce SB and SBB

s
